# A Novel Task Trainer for Auricular Hematoma Repair

**DOI:** 10.7759/cureus.21600

**Published:** 2022-01-25

**Authors:** Ryan Walsh, Tracy Fennessy, Emily Pauw, Michael Lajeunesse, Kyle Couperus

**Affiliations:** 1 Department of Emergency Medicine, Vanderbilt University Medical Center, Nashville, USA; 2 Department of Emergency Medicine, Madigan Army Medical Center, Tacoma, USA

**Keywords:** task trainer, emergency medicine, emergency medicine training, emergency medicine procedural competency, auricular hematoma drainage, auricular hematoma

## Abstract

Auricular hematoma drainage is a crucial skill that emergency medicine providers must be proficient in to prevent complications including permanent deformity of the ear. We aimed to develop and evaluate a cost-effective task trainer to allow emergency medicine (EM) residents to practice the key skills of auricular hematoma drainage and pressure dressing application.

After creating a task trainer out of a bell pepper, we implemented this training during our EM simulation conference with a total of 20 PGY 1-3 EM residents. Prior to the simulation session, a survey of all 39 residents found a rated confidence level of auricular hematoma drainage as low on a five-point Likert scale (mean: 2.2 (standard deviation (SD): 1.08)). After the session, the 20 EM residents who participated were much more confident in their ability to perform this procedure (mean: 4.4 (SD: 0.6)).

This low-cost, easy-to-create auricular hematoma drainage and repair task trainer was well received by our EM residents and led to an improved resident comfort level in performing this necessary EM procedure. This task trainer can be used by EM trainees of all levels as a tool to increase their skill and comfort level when performing the crucial procedure of auricular hematoma drainage and repair.

## Introduction

An auricular hematoma is a collection of blood between the cartilage and perichondrium of the ear that typically results from blunt trauma, particularly from contact sports including wrestling and boxing. If not drained promptly and properly, this injury prevents perfusion of the underlying cartilage and can result in cartilage necrosis, fibrosis, and disorganized growth of new cartilage [1]. This results in a permanent deformity of the ear commonly referred to as the “cauliflower ear.” This is particularly common in athletes who do not wear headgear, as a survey of collegiate wrestlers found that they were twice as likely to develop an auricular hematoma and almost three times as likely to develop cauliflower ear when not wearing a helmet [2].

The treatment for an auricular hematoma is drainage as soon as possible after the injury, with the approach depending on the size and age of the hematoma. An extensive Cochrane review of the existing literature on auricular hematoma treatment found 48 articles detailing multiple methods of repair, including needle aspiration, incision and evacuation, and anterior or posterior incisions. However, there was no evidence to conclude that one intervention was superior to another [3]. General guidelines suggest that a hematoma less than seven days old may be drained by either needle aspiration (if <2 cm and <48 hours old) or incision and drainage (if ≥2 cm or >48 hours old) in the emergency department [1,3-5]. A hematoma greater than seven days old may have already begun to fibrose and warrants a referral to a specialist such as an otolaryngologist or plastic surgeon.

Following drainage, an appropriate pressure dressing must subsequently be applied to reduce the likelihood of recurrence. Options for pressure dressings vary widely and include bolster dressings, silicone splints, and even a swimmer’s nose clip [2]. Two studies from an otolaryngology journal found “bolsterless” repair using mattress sutures to be well tolerated and successful in preventing reaccumulation of the hematoma [6,7].

Regardless of the specific technique, auricular hematoma drainage and pressure dressing application are crucial skills that emergency medicine (EM) providers must be proficient in performing. These skills allow for prompt restoration of perfusion to the injured cartilage and greatly reduce the likelihood of permanent disfiguration of the ear.

We believe this is the first published description of an auricular hematoma drainage task trainer. We present an easily made and cost-effective task trainer that allows EM providers to practice these key skills of performing auricular hematoma drainage and pressure dressing/bolster application. This model was previously presented as a poster in January 2020 at the International Meeting on Simulation in Healthcare in San Diego, California.

## Technical report

**Figure 1:**
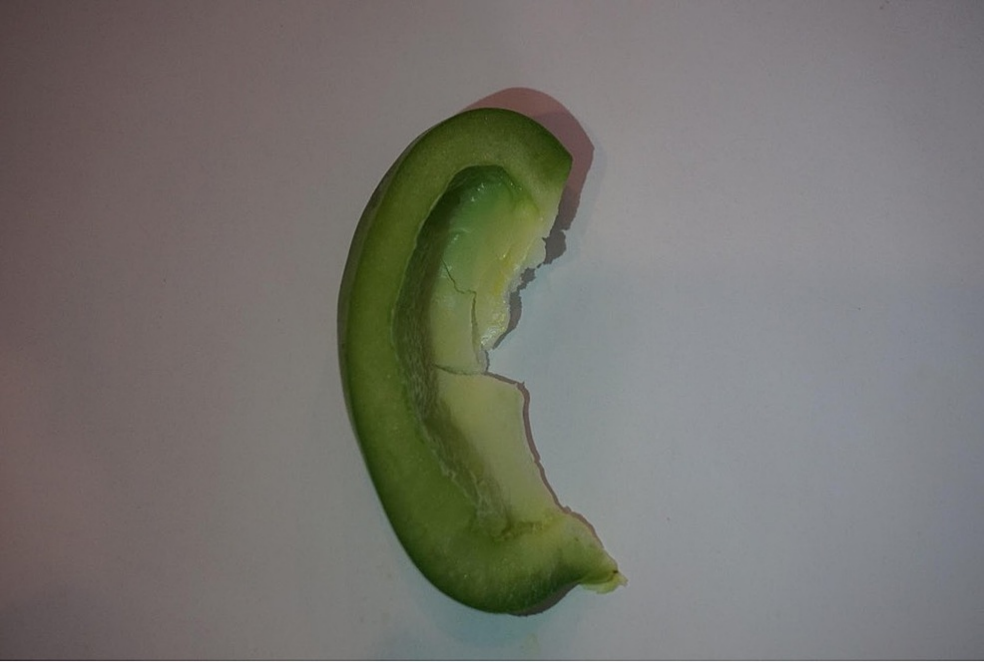
A slice of bell pepper simulating the ear.

Our primary objective was to develop a task trainer made of readily available, low-cost materials that increased resident knowledge, confidence, and proficiency in auricular hematoma drainage and repair. We constructed the task trainer by slicing a bell pepper lengthwise, with a 2 cm slice representing the auricle of the ear (Figure 1).

**Figure 2:**
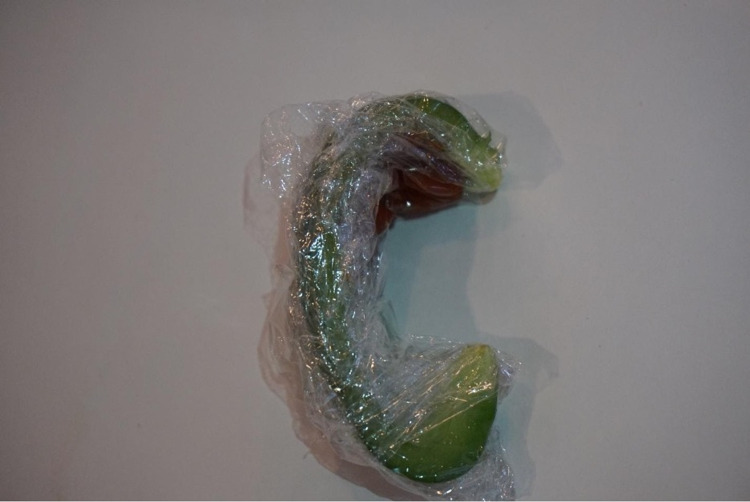
Bell pepper wrapped with a plastic wrap with ketchup injected along the simulated antihelix representing an auricular hematoma.

The bell pepper was then enveloped in a plastic wrap, and utilizing a 10 cc syringe and an 18-gauge needle, approximately 5 mL of ketchup was injected along the curvature of the bell pepper to produce a simulated hematoma (Figure 2).

**Figure 3:**
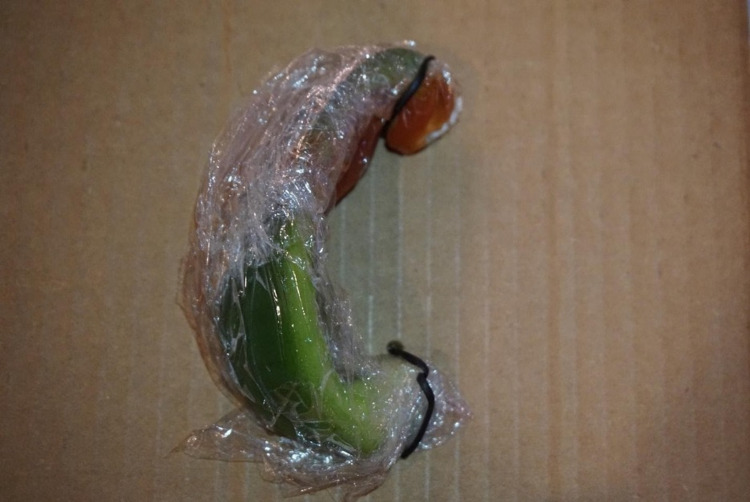
Twist ties utilized to secure the model to a piece of cardboard. The task trainer is now ready to be utilized.

**Figure 4:**
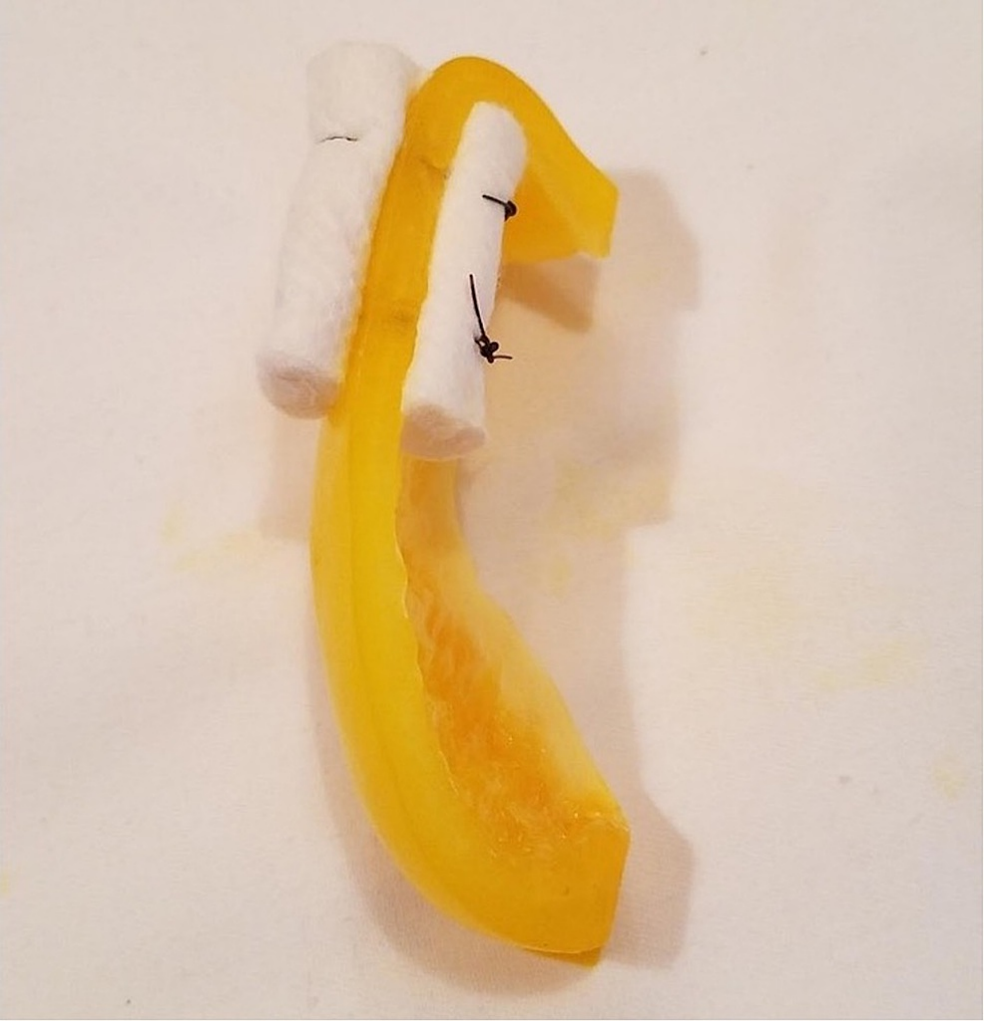
Example of task trainer utilized for bolster application.

To ensure the bell pepper model would not move during the drainage procedure, the bell pepper was attached to a piece of cardboard (Figures 3, 4).

Our model was implemented during our monthly EM simulation conference with participants from all three PGY levels. All participating EM residents were given a brief 10-minute presentation on auricular hematoma drainage and repair. Following this presentation, the participants utilized the task trainer for hands-on practice. This hands-on session lasted 30 minutes. Utilizing a five-point Likert scale, the participants rated the task trainer and their comfort with performing the procedure on anonymous surveys. We compared post-training comfort level to baseline comfort level that was obtained through a prior survey administered at the beginning of the previous academic year as part of a residency level needs assessment. The initial survey was administered to the entire EM residency (a total of 39 residents) to evaluate their comfort on a five-point Likert scale performing a total of 79 core EM procedures (including auricular hematoma drainage). This initial survey of resident comfort level with auricular hematoma drainage was used as a baseline for comparison to comfort level after the implementation of our task trainer. This project was considered exempt by the institutional review board.

## Discussion

A total of 20 EM residents participated in the procedural session, and all participants completed the anonymous post-training survey. When asked if they agreed that the task trainer model utilized was adequate to learn the necessary procedural skills, all residents rated the model very highly (mean: 5 (standard deviation (SD): 0)). Prior to the simulation session, as part of a separate residency level needs assessment, a survey of all 39 residents found that they rated their confidence level in performing auricular hematoma drainage as low (mean: 2.2 (SD: 1.08)) (Table 1). After the session, the 20 EM residents who participated in the session were much more confident in their ability to perform this procedure (mean: 4.4 (SD: 0.6)) (Table 1). Over half the participants (11) provided qualitative comments stating that the task trainer was realistic and that utilizing this task trainer provided additive benefit to the didactic with regard to the overall training activity.

**Table 1:** Assessment of the task trainer by the residents using a five-point Likert scale SD: standard deviation Performed using a five-point Likert scale: 1 = strongly disagree, 2 = disagree, 3 = neither agree nor disagree, 4 = agree, 5 = strongly agree

	Number of residents	“I feel confident performing auricular hematoma drainage” (SD)	“Task trainer adequate to learn necessary procedural skills” (SD)
Pre-training survey	39	2.2 (1.08)	-
Post-training survey	20	4.4 (0.6)	5 (0)

The strengths of the model include the ease of creation, low cost, and ability to prepare for multiple learners ahead of the training session to facilitate a quick transition between learner groups. One limitation of this model is that the plastic wrap does not create a tight “neat” fit over the bell pepper, which reduces some anatomic fidelity of the hematoma. An additional limitation is that the initial comfort survey included all 39 residents, while the post-training session survey included only 20 EM residents. This could have impacted the overall difference between the pre- and post-comfort levels; however, we suspect that this difference would have been minimal as the standard deviations of the pre- and post-comfort levels were small.

## Conclusions

Auricular hematoma drainage is a crucial emergency medical procedure to prevent complications including permanent deformity of the ear. Despite this, to our knowledge, there are no prior published descriptions of auricular hematoma drainage task trainers. This low-cost, easy-to-create auricular hematoma drainage and repair task trainer was well received by our EM residents and led to an improved resident comfort level in performing this necessary EM procedure. We believe that this task trainer can be used by EM trainees of all levels as a tool to increase their skill and comfort level with performing the crucial procedure of auricular hematoma drainage and repair.
